# Evidence of Infection with H4 and H11 Avian Influenza Viruses among Lebanese Chicken Growers

**DOI:** 10.1371/journal.pone.0026818

**Published:** 2011-10-28

**Authors:** Ghazi Kayali, Elie Barbour, Ghassan Dbaibo, Carelle Tabet, Maya Saade, Houssam A. Shaib, Jennifer Debeauchamp, Richard J. Webby

**Affiliations:** 1 Division of Virology, Department of Infectious Diseases, St. Jude Children's Research Hospital, Memphis, Tennessee, United States of America; 2 Faculty of Agricultural and Food Sciences, American University of Beirut, Beirut, Lebanon; 3 Faculty of Medicine, American University of Beirut, Beirut, Lebanon; Duke-NUS Graduate Medical School, Singapore

## Abstract

Human infections with H5, H7, and H9 avian influenza viruses are well documented. Exposure to poultry is the most important risk factor for humans becoming infected with these viruses. Data on human infection with other low pathogenicity avian influenza viruses is sparse but suggests that such infections may occur. Lebanon is a Mediterranean country lying under two major migratory birds flyways and is home to many wild and domestic bird species. Previous reports from this country demonstrated that low pathogenicity avian influenza viruses are in circulation but highly pathogenic H5N1 viruses were not reported. In order to study the extent of human infection with avian influenza viruses in Lebanon, we carried out a seroprevalence cross-sectional study into which 200 poultry-exposed individuals and 50 non-exposed controls were enrolled. We obtained their sera and tested it for the presence of antibodies against avian influenza viruses types H4 through H16 and used a questionnaire to collect exposure data. Our microneutralization assay results suggested that backyard poultry growers may have been previously infected with H4 and H11 avian influenza viruses. We confirmed these results by using a horse red blood cells hemagglutination inhibition assay. Our data also showed that farmers with antibodies against each virus type clustered in a small geographic area suggesting that unrecognized outbreaks among birds may have led to these human infections. In conclusion, this study suggests that occupational exposure to chicken is a risk factor for infection with avian influenza especially among backyard growers and that H4 and H11 influenza viruses may possess the ability to cross the species barrier to infect humans.

## Introduction

Avian influenza virus transmission to humans has increased since the first documented case that occurred in Hong Kong during 1997 [1]. Since that time, avian-to-human influenza virus transmission has been documented in many nations [2]. The most recent avian influenza infections in humans have involved H5N1 strains. These viruses have caused at least 562 human illnesses and 329 deaths (59% mortality) since January 2003 [3].

Exposure to poultry infected with highly pathogenic avian influenza (HPAI) H5 viruses is the most important risk factor for humans becoming infected with HPAI H5 viruses as suggested by research in China, Vietnam, and Thailand [4], [5], [6], [7], [8], [9], [10]. A case series of Turkish patients revealed that all of the 8 H5N1 infected patients had a history of contact with ill or dead chickens [11]. HPAI viruses of the H7 subtype are also capable of infecting humans. In February 2003, an outbreak of HPAI H7N7 affected poultry in the Netherlands. Studies related to this outbreak showed that poultry workers and their household contacts had evidence of infection with the same virus [12], [13], [14]. An outbreak of an H7N3 virus in Canadian poultry left a culler and another poultry worker with confirmed H7N3 infection [15].

There is also evidence of human infection with low pathogenic avian influenza (LPAI) viruses in areas where HPAI viruses are not present. In the US, studies among farmers, veterinarians, meat processing workers, hunters, wildlife biologists, poultry workers, and swine workers, showed that these were occupations at risk for zoonotic influenza infections [16], [17], [18], [19]. In a prospective study of 803 farmers in the US Midwest, there was serologic evidence of previous infection with LPAI virus types H5, H6, and H7 among farmers who had exposure or direct contact with live poultry or among participants who hunted wild birds [20]. In another study, researchers found cross-sectional evidence of previous infection with these same 3 virus subtypes among veterinarians who work with poultry [21]. Furthermore, researchers studied the sera of wildlife professionals and duck hunters and found 3 subjects with elevated antibody titers against an avian H11 influenza virus [16]. Most recently, evidence of LPAI H4, H5, H6, H9, and H10 virus infections was found among workers exposed to turkeys in small or free-ranging turkey farms [22].

Lebanon is in the heart of the Middle East surrounded by countries that reported the presence of HPAI H5 viruses in their poultry and human populations. Furthermore, Lebanon lies under two major wild bird migratory routes, the Mediterranean-Black Sea route and the West Asia-Africa route. Thus, Lebanon's geographic location increases the possibility of introducing AI viruses to domestic poultry flocks by migrating birds shedding these viruses. The literature carries very sparse studies on human cases of avian influenza in Lebanon and the Middle East. In a recent study, Lebanese researchers reported that 32.3% of individuals exposed to poultry infected with LPAI H9 viruses show elevated antibody titers against viruses of the same subtypes [23]. Here we conducted a controlled, cross-sectional, seroepidemiological study with the aim of measuring antibodies against LPAI viruses among Lebanese chicken growers and non-chicken exposed controls and determining associated risk factors.

## Materials and Methods

### Subjects

Between July and September 2010, we enrolled 200 chicken exposed and 50 non-exposed individuals. According to our sample size calculations using Epi Info v.3.5.1 software (CDC, Atlanta, GA), enrolling 89 exposed and 38 non-exposed subjects would have been sufficient to detect a 19% difference in prevalence among the two groups at 80% power and 95% confidence. Exposed individuals were identified and enrolled through agricultural cooperative associations from rural towns and villages of the Bekaa governorate (n = 94), North Lebanon governorate (n = 70), and South Lebanon governorate (n = 36). Growers were further classified by the type of agricultural practice that they practice, whether commercial or backyard. The non-exposed controls were enrolled from the capital Beirut and from the urbanized Mount Lebanon governorate (n = 50), areas where agriculture and poultry growing is not practiced and were invited to participate by word of mouth. Exclusion criteria were self-reported being less than 18 years of age, having and immunosuppressive illness or taking immunosuppressive therapy, or had exposure to poultry (if enrolled as a control). Study participants were interviewed face-to-face by a study staff member using a questionnaire that included demographic, occupational, and general health questions. We asked the growers about their use of vaccines for their poultries. A phlebotomist obtained a blood specimen for laboratory analysis. The blood was allowed to clot at room temperature then centrifuged on the same day of collection. Serum specimens were aliqouted into multiple cryovials, labelled and preserved at −20°C until ready for laboratory study. Serological studies were performed at the St. Jude Children's Research Hospital influenza laboratories, Memphis, TN, USA after sera were shipped over dry ice from Beirut, Lebanon. All participants completed the study interview and blood was successfully obtained from 248 of 250 participants. This study was approved by the institutional review boards of the American University of Beirut and St. Jude Children's Research Hospital. All subjects signed informed consent documents.

### Microneutralization (MN) Assay

A MN assay was used as the main assay to test sera for antibodies against avian influenza viruses. The assay's procedure is described elsewhere [16] and was adapted from Rowe et. al [24]. As LPAI viruses from Lebanon were not available, we selected a panel of avian viruses that cross-react widely with panels of antisera prepared against their relative hemagglutinin types (data not shown). These viruses, listed in Table 1, were form Eurasian origins except for North American H8 and H12 viruses and an Australian H15 virus. Sera were tested in duplicate and were considered positive if titers were positive at ≥1∶10 dilutions. We used this low threshold of evidence of infection as have others [25], as there is evidence from recent trials of human H5N1 vaccines showing that neutralizing antibodies against the vaccine strains drop nearly to pre-vaccination titers over a period of time as short as 6 months [26], [27].

**Table 1:** Viruses used in the microneutralization and hemagglutination inhibition assays.

Virus Name	Subtype
A/duck/Hong Kong/365/78	H4N6
RG-A/turkey/Egypt/7/2007	H5N1
A/quail/Hong Kong/YU 421/02	H6N1
RG-A/Netherlands/219/2003	H7N7
A/turkey/Ontario/6118/68	H8N4
A/turkey/Israel/1567/04	H9N2
A/chicken/Germany/N/49	H10N7
A/duck/Hong Kong/P50/97	H11N9
A/duck/Alberta/60/76	H12N5
A/gull/Astrachan/458/85	H13N6
A/mallard duck/Astrachan/263/82	H14N5
A/wedge-tailed shearwater/Western Australia/2576/79	H15N9
A/black-headed gull/Sweden/5/99	H16N3
A/Brisbane/59/04	H1N1
A/California/04/09	H1N1
A/Brisbane/10/07	H3N2

### Hemagglutination Inhibition (HI) Assays

Horse blood HI was used as an alternative assay to test sera that were positive by MN for the presence of antibodies against avian influenza viruses. In order to rule out potential cross-reactivity between antibodies against human influenza viruses and avian influenza viruses, we tested the sera for antibodies against human seasonal and pandemic influenza viruses using a turkey blood HI assay. Horse blood (Rockland, Gilbertsville, PA) was washed three times with PBS by mixing 20 ml of blood and 30 ml of PBS in a 50 ml centrifuge tube and centrifuging at 4°C, 1000×g for 5 minutes. A solution of 1% horse blood was prepared by adding pelleted horse RBCs to PBS containing 0.5% bovine serum albumin fraction V. Turkey blood (Rockland) was washed three times, and a solution of 0.5% turkey blood was prepared by adding pelleted turkey RBCs to PBS.

Sera were treated with receptor destroying enzyme (RDE) (Denka Seiken, Tokyo, Japan) by mixing one part sera to 3 parts RDE, incubating overnight at 37°C, then heat inactivated at 56°C for 30 minutes. Sera were further diluted with PBS to a final 1∶10 dilution. Two-fold serial dilutions in 25 µl PBS were performed. Next, 25 µl of PBS containing 4 hemagglutination units (HAU) of virus was added to each serum dilution. The sera and virus mixture was incubated at room temperature for 30 minutes after which 50 µl of 1% horse blood or 0.5% turkey blood was added to each well. Plates were incubated at room temperature and read after 30 minutes for turkey blood and 1 hour for horse blood. The serum HI titer result was expressed as the reciprocal of the highest dilution of serum where hemagglutination was inhibited.

### Statistical Methods

Pearson's Chi square and Fisher's exact tests were used to compare categorical variables. Student's t-test was used to compare continuous variables with normal distributions. Mood's median test for independent samples was used to compare medians for continuous variables that were not normally distributed. Geometric mean titers were calculated for each influenza subtype under study and titers were compared by using the Kruskal Wallis test. A p-value less than 0.05 was considered significant. Analysis was performed using the PASW (SPSS) 18.0 software.

## Results

Backyard chicken growing was practiced by 128 growers who kept seasonal flocks of chicken numbering less than 100 birds. The remaining 72 farmers raised larger flocks of chicken for commercial purposes. Data in Table 2 show the demographic characteristics of the study subjects. Chicken growers were significantly older and more likely to be of the male sex than controls. Backyard growers were less educated than farmers and controls respectively (p-value = 0.014). Use of tobacco products was more frequent among commercial growers (71%) as compared to backyard growers and controls (40%). There was a significant difference among the groups for using seasonal influenza vaccine. More controls (30%) reported receiving the seasonal influenza vaccine in the previous influenza season than chicken growers (<10%). Among all groups, there was no significant difference in the reporting of influenza-like illness (ILI) in the past 12 months prior to enrollment or chronic conditions including cancer, heart disease, diabetes, and other conditions that affect the immune system.

**Table 2:** Distribution of demographic and health variables among study groups.

Variable		Controls (n = 50)	Backyard Growers (n = 128)	Commercial Farmers (n = 72)	p-value
Age	Mean Age (SD)	37.6 (11.8)	44.4 (16.1)	40.0 (14.7)	**0.014**
Gender	Male	15 (30.0)	58 (45.3)	60 (83.3)	**<0.001**
	Female	35 (70.0)	70 (54.7)	12 (16.7)	
Educational Level	None/Elementary	4 (8.0)	75 (58.6)	35 (48.6)	**<0.001**
	Intermediate	11 (22.0)	25 (19.5)	23 (31.9)	
	Secondary/College	9 (18.0)	20 (15.6)	9 (12.5)	
	Graduate Degree	26 (52.0)	8 (6.3)	5 (6.9)	
Use Tobacco Products	Yes	20 (40.0)	51 (39.8)	51 (70.8)	**<0.001**
	No	30 (60.0)	77 (60.2)	21 (29.2)	
Chronic Disease	Yes	5 (10.0)	18 (14.1)	7 (9.7)	0.559
	No	45 (90.0)	110 (85.9)	65 (90.3)	
Influenza-like Illness	Yes	20 (40.0)	46 (35.9)	19 (26.4)	0.205
	No	30 (60.0)	82 (64.1)	53 (73.6)	
Influenza Vaccine	Yes	15(30.0)	9(7.0)	3(4.2)	**<0.001**
	No	35(70.0)	119(93.0)	69(95.8)	

P-values in bold are significant. For age, numbers indicate mean and standard deviation (SD); for all other variables, numbers indicate N(%).

We then explored the exposure profiles of the study subjects (Table 3). The median number of years of working with chicken was similar in both exposure groups. The median number of days since last contact with chicken was zero. Commercial farmers were significantly exposed to more chicken (median = 2000 birds) and spent more hours per week (median = 21 hours) working with birds than backyard growers. Commercial growers were also more likely to use protective masks, footwear, and clothes than backyard growers. There was no difference in the use of eye protection and gloves between the two groups. We detected a significant difference in exposure to turkeys between the two groups, but no difference in exposure to other domestic or wild birds or pigs. The use of poultry vaccines was more practiced by commercial growers (71%) than backyard growers (9%). The most commonly used vaccines were against H9 avian influenza and Newcastle disease virus. Similar rates of dead or sick chickens were reported by both groups (35–45%); however reason of death or illness was not ascertained.

**Table 3:** Distribution of exposure variables among study exposed groups.

Variable	Backyard Growers (n = 128)	Commercial Farmers (n = 72)	P-value
Median Years Working with Chickens	6.0(3–15)	5.5(2–10.5)	0.837
Median Days since last Contact	0(0-0)	0(0-0)	-
Median number of Chickens	14(9–30)	2000(400–7250)	**<0.0001**
Median work hours/week	3.5(1.75–7)	21.0(7–56)	**<0.0001**
Proper Use of Mask	0(0.0)	4(5.5)	**0.015**
Proper Use of Footwear	8(6.3)	11(15.3)	**0.037**
Proper Clothing	0(0.0)	3(4.2)	**0.045**
Proper Eye Protection	5(3.9)	2(2.9)	0.526
Proper Use of Gloves	2(1.6)	1(1.4)	0.936
Exposed to Livestock	46(35.9)	20(27.8)	0.276
Exposed to Turkeys	4(3.1)	9(12.5)	**0.015**
Exposed to Ducks, geese, quails	15(11.7)	14(19.4)	0.121
Exposed to Wild birds	7(5.5)	9(12.5)	0.071
Exposed to Pigs	0(0.0)	0(0.0)	-
Vaccinate Chickens	12(9.4)	51(70.8)	**<0.001**
Exposed to Sick/Dead Chickens	45(35.2)	33(45.8)	0.185

P-values in bold are significant. Medians are presented with their interquartile ranges in parentheses. For all other variables, numbers indicate N(%).

We used turkey blood HI to test for antibodies against human influenza viruses. The control group had significantly higher titers against seasonal and pandemic influenza H1N1 but there was no difference in antibody titers against seasonal H3N2 viruses (Table 4).

**Table 4:** Distribution of turkey red blood cells hemagglutination inhibition antibody titers against human influenza viruses among study groups.

Influenza Virus	Titer	Controls (n = 50)	Backyard Growers (n = 128)	Commercial Farmers (n = 72)	p-value
Seasonal H1	<1∶40	3(6.0)	116(92.1)	61(88.4)	**0.003**
	1∶40	5(10.0)	8(6.3)	3(4.3)	
	1∶80	37(74.0)	1(0.8)	5(7.2)	
	1∶160	3(6.0)	1(0.8)	0(0)	
	1∶320	1 (2.0)	0(0)	0(0)	
	1∶640	1 (2.0)	0(0)	0(0)	
	GMT	29.90	21.48	22.79	
Pandemic H1	<1∶10	47(94.0)	126(100.0)	66(95.7)	**0.034**
	1∶10	0(0)	0(0)	0(0)	
	1∶20	1(2.0)	0(0)	1(1.4)	
	1∶40	2(4.0)	0(0)	0(0)	
	1∶80	0(0)	0(0)	2(2.9)	
	GMT	5.59	5.00	5.53	
Seasonal H3	<1∶40	34(68.0)	74(58.7)	34(49.3)	0.336
	1∶40	3(6.0)	5(4.0)	9(13.0)	
	1∶80	3(6.0)	17(13.5)	11(15.9)	
	1∶160	7(14.0)	12(9.5)	8(11.6)	
	1∶320	1(2.0)	12(9.5)	3(4.3)	
	1∶640	1(2.0)	3(2.4)	3(4.3)	
	1∶1280	0(0)	2(1.6)	1(1.4)	
	1∶2560	1(2.0)	1(0.8)	0(0)	
	GMT	37.84	47.44	45.56	

P-values in bold are significant. GMT indicates Geometric Mean Titer. Numbers indicate N(%).

Five of the backyard growers had elevated antibody titers against LPAI viruses (Table 5). No titers were detected among commercial farmers or controls. Three of these individuals tested positive for H4 antibodies by MN. The titers were 1∶10, 1∶80, and 1∶160. All three individuals were males from the Baalbek district in Bekaa governorate in the Northeastern part of Lebanon. The other two individuals were positive against H11 and the titers were 1∶20 and 1∶80. Both of these subjects (a male and a female) came from the Tyre (Sour) district in the South Lebanon governorate. We used horse RBC HI to test the sera of these five subjects and that of subjects who tested negative for antibodies against LPAI by MN. When H4 was used as antigen, the three subjects who tested positive by MN were positive by horse RBC HI and the titers were between 1∶10 and 1∶40. The other sera tested negative. The same findings were obtained when H11 was used as antigen. Both subjects who were positive by MN remained positive by horse RBC HI (1∶40 and 1∶20), and the other sera were negative. We compared the backyard growers who tested positive for any LPAI virus to those who tested negative. We found no significant difference in demographic, health, or exposure variables.

**Table 5:** Antibody titers against avian influenza viruses among backyard chicken growers.

Virus	Subject ID	Microneutralization Titer	Horse Blood HI Titer	Governorate/District	Gender	Year of Birth
Avian H4	86	1∶10	1∶10	Bekaa/Baalbek	Male	1991
	89	1∶80	1∶10	Bekaa/Baalbek	Male	1972
	90	1∶160	1∶40	Bekaa/Baalbek	Male	1943
Avian H11	23	1∶20	1∶40	South Lebanon/Sour	Male	1952
	14	1∶80	1∶20	South Lebanon/Sour	Female	1978

## Discussion

In this study, we provide evidence suggesting that occupational exposure to chickens potentially infected with LPAI viruses increases the risk of infection with these viruses in Lebanon. This finding was among backyard growers and not commercial farmers. To our knowledge, this is the only study that has investigated infection with a wide range of AI viruses among individuals exposed to chicken in Lebanon.

In order to verify our serological findings, we retested the sera found positive by MN using a horse RBC HI assay and found consistent results. The geographic clustering of subjects testing positive for the same virus, as well as consistency of results obtained by different assays, strengthens the evidence as these individuals may have been exposed to chicken infected with H4 or H11 viruses in the areas where they work.

Small numbers of subjects were seropositive for LPAI viruses and this could have affected results of the subgroup analyses and might explain our inability to detect significant risk or protective factors associated with infection. However, the backyard farmers were less likely to utilize protective equipment and thus may be at higher risk of infection. Higher rates of using poultry vaccines and protective equipment among commercial farmers may be indicative of slightly better biosecurity measures in commercial farms thus protecting the workers at such establishments. Around 40% of the poultry growers reported exposure to sick or dead poultry. However, we were not able to determine the exact cause of death or illness of the poultry. We were also not able to specify avian influenza disease distribution among poultry in Lebanon. Furthermore, from our data and although a remote possibility, it is not possible to rule out human-to-human transmission.

This is not the first time that possible infection with H4 and H11 viruses among individuals exposed to poultry and wild birds is reported. The odds ratio of infection with H4 viruses among backyard turkey growers in the US was 3.9 (95% CI: 1.2–12.8) as compared to non-exposed controls [28]. In another study, one duck hunter and two wildlife professionals in the US were found to be seropositive for H11 antibodies [16]. Our findings among commercial farmers support similar evidence among large scale poultry growers. In a study of workers in poultry confinement farms in Peru , there was no indication of infection with AI subtypes H4-H12 [29]. Similarly, there was no evidence of infection with H5 among Nigerian poultry workers [30]. Studying the patterns of antibody cross-reactivity between human and avian influenza viruses was beyond the objectives of this study. Although possible, we do not believe that cross-reactivity of antibodies against human influenza viruses with avian influenza viruses may explain our findings. All subjects with positive antibody titers against avian influenza viruses had low titers of 1∶20 against a seasonal H1N1 virus and negative titers against a pandemic H1N1 virus. Two subjects with antibodies against the H4 subtype had a titer of 1∶320 against the seasonal H3N2 virus, while one subject with antibodies against the H11 subtype tested positive for antibodies against H3N2 (titer = 1∶80). These subjects did not have the highest titers against H3N2. Thus, there is no clear cross-reactivity pattern between antibodies against H3N2 and the H4 and H11 subtypes. One individual with antibody titer against H4 and another person with a titer against H11 were alive when the human H2N2 viruses circulated, hence cross-reactivity of titers against an H2 subtype can also be ruled out.

We were not able to find data on avian influenza viruses in poultry in Lebanon other than sporadic reports of cross-sectional surveys or outbreak investigations [23], [31]. Lack of systematic monitoring would delay preventative interventions and outbreak control measures especially if highly pathogenic viruses emerge. This would not only affect poultry but also farmers who are exposed to these animals. Lebanon and other developing countries should increase their efforts on monitoring avian influenza in poultry and research at the animal human interface. In conclusion, this study adds to the evidence that occupational exposure to chickens potentially infected with avian influenza is a risk factor for infection with AI especially among backyard growers.
